# Molecular Mechanisms and Clinical Divergences in HPV-Positive Cervical vs. Oropharyngeal Cancers: A Critical Narrative Review

**DOI:** 10.1186/s12916-025-04247-z

**Published:** 2025-07-07

**Authors:** Canio Martinelli, Alfredo Ercoli, Silvana Parisi, Giuseppe Iatì, Stefano Pergolizzi, Luigi Alfano, Francesca Pentimalli, Michelino De Laurentiis, Antonio Giordano, Salvatore Cortellino

**Affiliations:** 1 Unit of Gynecology and Obstetrics, Department of Human Pathology of Adults and Developmental Age, G, Martino University Hospital, Messina, Italy; 2 Sbarro Institute for Cancer Research and Molecular Medicine and Center for Biotechnology, College of Science and Technology, Temple University, Philadelphia, PA USA; 3 Radiation Oncology Unit, Department of Biomedical, Dental Science and Morphological and Functional Images, University of Messina, Messina, Italy; 4 Cell Biology and Biotherapy Unit, Istituto Nazionale Tumori -IRCCS-Fondazione G. Pascale, Naples, Italy; 5 Department of Medicine and Surgery, LUM University Giuseppe De Gennaro, Casamassima, Bari, Italy; 6 Division of Breast Medical Oncology, Istituto Nazionale Tumori -IRCCS-Fondazione G. Pascale, Naples, Italy; 7 Department of Medical Biotechnologies, University of Siena, 53100 Siena, Italy; 8 Laboratory of Molecular Oncology, Responsible Research Hospital, Campobasso, Italy; 9 Clinical and Translational Oncology, Scuola Superiore Meridionale, Naples, Italy; 10 SHRO Italia Foundation ETS, Candiolo, Turin, Italy

**Keywords:** Human papillomavirus, Cervical cancer, Oropharyngeal cancer, Molecular oncology, Radiosensitivity

## Abstract

Human papillomavirus (HPV) plays a pivotal role in the development of both cervical squamous cell carcinoma (CSCC) and oropharyngeal squamous cell carcinoma (OPSCC). However, these two cancers exhibit markedly different clinical behaviors. While HPV-positive OPSCC is distinguished by its heightened radiosensitivity, enabling effective treatment de-escalation and reduced toxicity, HPV-positive CSCC shows no such advantage, requiring aggressive therapeutic approaches similar to HPV-negative cases. This critical narrative review explores the limited molecular drivers currently known and the potential mechanisms underlying the divergent clinical responses of HPV-positive OPSCC and CSCC. Here, we discuss the role of HPV E6 and E7 oncoproteins in disrupting key tumor suppressor pathways, the impact of HPV DNA integration into the host genome, and the resulting genomic instability. By comparing the molecular mechanisms of these cancers, we aim to provide a comprehensive understanding of how these processes contribute to their distinct radiosensitivities and clinical outcomes. This review further highlights the gaps in the current research and proposes areas for future investigation, particularly in tailoring personalized treatment strategies for HPV-driven cancers. Understanding the differences in the molecular pathways that influence radiosensitivity in HPV-related cancers will not only enhance treatment strategies but also lead to improved patient outcomes and reduced treatment-associated toxicity.

## Background

Human papillomavirus (HPV) contributes to approximately 5% of the global cancer burden [1]. Of the 200 + HPV genotypes, only a few (mainly HPV-16 and HPV-18) are highly carcinogenic and sexually transmitted. These genotypes are responsible for nearly all cervical squamous cell carcinomas (CSCC) and a rising proportion of oropharyngeal squamous cell carcinomas (OPSCC) [2]. In the United States, an estimated 13,820 new cases of invasive cervical cancer and 4,360 related deaths occur each year [3]. Similarly, 58,450 new cases of oral cavity and oropharyngeal cancers are expected, with 12,230 associated deaths [4]. HPV accounts for approximately 70% of all OPSCC cases. The incidence is higher in men (particularly those aged 20–44 years) where the male:female ratio may reach 3:1 [5–7].

HPV infection drives oncogenesis in both CSCC and OPSCC; however, despite this shared viral etiology, the clinical behavior and treatment responses of these cancers differ markedly. HPV-positive OPSCC is more radiosensitive than its HPV-negative counterpart, leading to better treatment outcomes and improved survival rates [8]. In contrast, HPV-positive CSCC does not exhibit increased radiosensitivity, requiring the same aggressive treatment protocols used for HPV-negative disease [9, 10]. This clinical difference is so pronounced that the UICC/AJCC 8th edition TNM classification reflects the enhanced radiosensitivity and survival benefit of HPV-positive OPSCC by assigning lower stage categories compared to HPV-negative cases. Conversely, HPV status does not impact staging or treatment strategies for CSCC, which remains comparable to HPV-negative cases [9, 11, 12]. So, why does HPV confer radiosensitivity in OPSCC but not in CSCC? Exploring this issue is essential for enhancing our understanding of these malignancies and optimizing HPV-targeted treatment approaches in clinical practice. This review aims to delineate and synthesize the few molecular drivers known to be associated with OPSCC and CSCC, potentially elucidating their divergent clinical outcomes and treatment responses. To address the knowledge gap regarding molecular differences that may underlie variations in radiotherapy response, we will examine HPV-mediated tumorigenesis mechanisms. Our investigation will focus particularly on DNA repair mechanisms, which may be responsible for genetic and genomic alterations potentially accounting for the clinical distinctions between cervical and oropharyngeal cancers. By scrutinizing the altered biological processes in these tumors, we intend to elucidate the molecular basis of these differences and identify areas where the current understanding is insufficient. This approach may provide opportunities for further investigation, including Phase 0 studies, to yield novel insights. This molecular analysis of HPV-driven oncogenesis will inform future research directions and clinical strategies tailored to the specific biological profiles of OPSCC and CSCC with the objective of improving patient outcomes.

### HPV replication: hijacking the host cell cycle

**Fig. 1:**
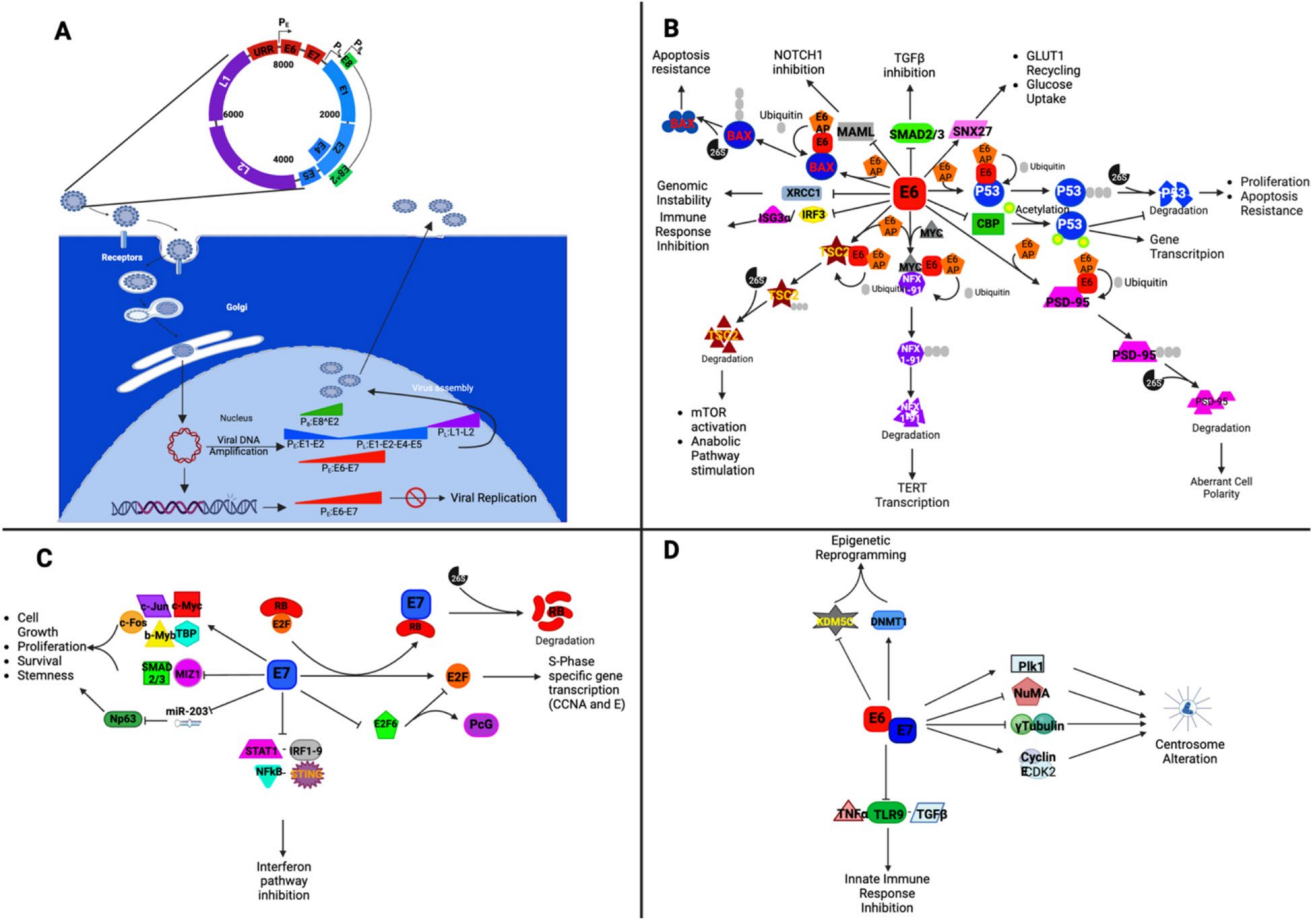
Schematic illustration of human papillomavirus (HPV) infection stages and oncogenic pathways altered by E6 and E7 oncoproteins. **A)** The virion binds to primary heparan sulfate proteoglycan receptors (HSPG1) in the ECM or cell surface and is transported along actin-rich protrusions to secondary HSPG-binding sites (HSPG2). This induces conformational changes in the capsid, exposing the L2 amino-terminal for further cleavage, which dissociates the capsid from HSPGs and exposes an L1 binding site recognized by an entry receptor complex, triggering endocytosis. HPV particles are transported by early endosomes that mature into late endosomes upon lysosomal fusion. Acidic environments within lysosomes cause capsid disassembly, separating L1 from L2 and forming the viral genome-L2 complex. L2 transports the viral genome from the late endosome to the trans-Golgi network. During metaphase-anaphase, L2-containing Golgi-derived vesicles interact with microtubules, transporting viral DNA-containing vesicles to the nucleus. Translated early gene products, such as E2, bind viral DNA to mitotic chromosomes, granting access to cellular transcription and replication machinery, facilitating viral DNA establishment and maintenance in dividing cells. Early gene transcription facilitates viral DNA replication, which is later enveloped by L1 and L2 protein, transported across cytoplasmic membranes, and released extracellularly. HPV DNA may integrate into the host genome, disrupting the E2 open reading frame (ORF) and resulting in overexpression of viral oncoproteins regulated by the PE promoter. This integration can create fusion transcripts between the viral early region and the host gene polyadenylation site, enhancing the stability of E6 E7 bicistronic mRNA and increasing oncoprotein levels. Furthermore, viral DNA integration can induce insertional mutagenesis of host genes, potentially leading to oncogenic effects. HPVs circular double-stranded genome of HPV, approximately 8000 base pairs, is divided into three regions: early, late, and the non-coding upstream regulatory region (URR). The early region includes overlapping ORFs such as E1, E2, E4, E5, E6, E7, and E8. The late region contains ORFs L1 and L2, which encode the capsid proteins. HPV E2 protein and host factor-binding sequences within the URR regulate viral transcription and replication. The major promoters are PE, PL, and PE8. HPV genes are categorized by function and color: oncogenes E6 and E7 are red; other early genes E1, E2, E5, and E1^E4 are blue; the E8^E2 splicing variant is green; and capsid genes L1 and L2 are purple. **B)** The viral E6 protein promotes neoplastic cellular transformation by binding to specific proteins (XRCC1, IRF3, ISG3, MAML, SMAD2, SNX27, CBP) and facilitating the polyubiquitination and proteasomal degradation of others (p53, PSD95, NFX1-91, TSC2, BAX) through E6-AP ubiquitin ligase recruitment. This results in apoptosis resistance (BAX), genomic instability (XRCC1), altered immune response (IRF3, ISG3), anabolic pathway activation (mTORC2), telomere elongation (NFX1-91), disrupted cell polarity (PSD95), uncontrolled proliferation, transcriptional changes (p53), metabolic reprogramming (SNX27), and altered cell differentiation (MAML, TGFb). **C)** The oncogenic E7 protein enhances cell proliferation by activating E2F-regulated gene transcription through either RB degradation or E2F6 repressor inhibition. E7 promotes growth, proliferation, and stemness by activating (JUN, MYC, MYB, TBP) or inhibiting (SMAD2/3, MIZ1) transcription factors or by repressing miR-203 transcription. E7's interactions with STAT1, IRF1-9, NF-κB, and STING suppress the interferon pathway. **D)** E6 and E7 also alter centrosome formation by deregulating CyclinE/CDK2 and PLK1 activity, affecting γ-tubulin transport to centrosomes, and delocalizing NuMA, essential for mitotic spindle assembly. Additionally, they repress the innate immune response by interacting with TNFα, TGFβ, and TLR9 and induce epigenetic reprogramming by upregulating DNA methyltransferase DNMT1 and degrading lysine demethylase KDM5D in HPV-infected cells (created with BioRender.com)

The HPV16 replication cycle in cervical tissue has been the most extensively studied and will be outlined here. HPV infection in the oropharynx is assumed to follow a pattern similar to that in the cervix, although no studies have been conducted to date to confirm this hypothesis. The HPV genome is circular, approximately 8-kbp in size, and is divided into three major regions, encoding up to ten proteins (including spliced variants) that are essential for the virus. The"early"(E) region, approximately 4-kbp long, includes the open reading frames E1, E2, E4, E5, E6, E7, and E8, as well as the E8^E2 fusion transcript (derived by alternative splicing). These genes collectively drive early-phase processes, including viral DNA replication, regulation of the host cell cycle, and modulation of antiviral responses. The “late” (L) region, roughly 3-kbp, encodes the major and minor capsid proteins L1 and L2, which form the viral capsid. Finally, the upstream regulatory region (URR) (also called the long control region, LCR), approximately 1-kbp in length, contains promoters, enhancers, and the origin of replication critical for regulating viral DNA replication and transcription [13] (Fig. 1A). However, the viral genome does not encode all the proteins necessary for DNA replication, so it relies on the host cell replication machinery. To begin its reproductive cycle, HPV specifically targets the basal layer of epithelial cells, as these cells exhibit high proliferative activity, providing an ideal environment for the virus to replicate and establish persistence [14]. Furthermore, the HPV life cycle is closely linked to the differentiation program of the epithelial cells it infects. HPV enters these tissues through microlesions present in the external apical layer and is internalized by endocytosis.

Once viral DNA enters the nucleus, the initial phase of HPV replication begins with the expression of E2 protein, essential for initiating viral DNA replication and ensuring its segregation during cell division. E2 accomplishes this through two main mechanisms. First, E2 interacts with E1 helicase, which unwinds the viral DNA, enabling replication machinery to duplicate the genome. This step is crucial for starting the replication process. Second, E2 recruits transcriptional regulators to URR, promoting the expression of early genes necessary for viral replication under the control of the early promoter (PE). In addition to driving replication, E2 maintains the viral genome during cell division. E2 links viral DNA to the host mitotic chromosomes by interacting with proteins, such as BRD4 (an epigenetic reader), DDX11, and TOPBP1. This interaction ensures that the viral DNA is evenly distributed between daughter cells during mitosis, allowing the virus to persist as host cells divide [15–19].

At this stage, the virus needs to limit its replication to avoid overwhelming the host machinery and triggering an immune response. Unrestrained replication could lead to cell death, thereby eliminating the virus. The E8^E2 fusion protein, regulated by promoter P8, is key in this regulation because of its unique structure, created from an alternative mRNA splicing that fuses the E8 gene with exon 3′ of the E2 gene. Consequently, E8^E2 contains an N-terminal domain from E8 and a DNA-binding domain from E2. Although E8^E2 can homodimerize and heterodimerize with E2 and bind to the E2 DNA-binding sites in the viral URR, it lacks the ability to bind E1 helicase and many cellular transcriptional regulators normally recruited by the E2 N-terminal domain. This means that the E8^E2 fusion protein competes with E2 for the same DNA-binding sites in the URR; however, unlike E2, it cannot bind to E1 helicase, which is necessary for replication. By binding to E2 sites without activating replication, E8^E2 essentially"steals"E2’s role in promoting replication, thus regulating and limiting E2's activity [20–22].

As HPV-infected epithelial cells differentiate and migrate toward the suprabasal region, they enter the postmitotic phase. During this transition, the viral proteins E5, E6, and E7 are expressed, initiating significant reprogramming of the host cell genetic network, epigenetic landscape, and metabolism. Two pivotal actions of E6 and E7 stand out: the inhibition of key oncosuppressive proteins, such as p53 and RB, and activation of the MAPK signaling pathway. This reprogramming disrupts normal cellular functions, causing the blockade of differentiation, inhibition of apoptosis, repression of innate immune responses, and uncontrolled cell proliferation, while promoting late promoter activation [23, 24] (Fig. 1B-D). Consequently, there is increased expression of early viral proteins, such as E1 and E2, along with E4 and E5, and the transcription of L1 and L2 genes that code for capsid structural proteins, ensuring viral replication and assembly [23].

In the final stage of viral replication, HPV uses a sophisticated strategy to efficiently replicate while evading cell death and immune detection, ensuring long-term persistence in the host. The viral DNA, together with E1 and E7 proteins, activates the ataxia telangiectasia mutated (ATM) and ataxia telangiectasia and Rad3-related (ATR) kinases, key players in the DNA Damage Response (DDR) [25]. These proteins bind to viral DNA, facilitating the recruitment of replication machinery, while E4 promotes viral genome amplification by arresting the cell cycle in the G2 phase [26, 27]. Crucially, stalling in G2 ensures that viral replication avoids competition with host genomic DNA synthesis, which occurs during the S phase. This temporal separation allows the virus to amplify its genome by thousands of copies without interference, thereby ensuring efficient replication and persistence [28–30].

Once viral DNA is fully amplified, E2 binds to specific sites within the URR, halting further viral replication and the transcription of early viral genes responsible for cell cycle activation. By doing so, the virus effectively prevents the host cell from restarting division. This is crucial because resuming cell division would disrupt the assembly of viral particles and risk the dilution or loss of viral genomes, reducing the efficiency of virion production. Following this, changes in gene expression allow the translation of L1 and L2 capsid proteins, previously delayed by post-transcriptional regulation. These proteins are essential for packaging the viral DNA into the capsids [31, 32]. Finally, the virions are released from the epithelial surface into the extracellular spaces where they attach to cells via heparan sulfate proteoglycans (HSPGs), triggering a conformational change in capsid protein L1, termed “structural activation”, which facilitates the cleavage of L1 by a secreted protease, probably kallikrein-8 (KLK8). Subsequently, cyclophilin induces the externalization of the L2 N-terminal capsid protein from the capsid lumen. Furin cleavage of the L2 N-terminus reduces the capsid affinity for HSPGs and promotes interaction with a secondary receptor (integrin α6, annexin A2 heterotetramer, growth factor receptors, or tetraspanins CD63 and CD151) through which it is endocytosed. During its transport from the endoplasmic reticulum to the Golgi, the vesicles undergo changes that result in capsid removal and viral genome release. The genome then enters the nucleus through nuclear pores, where it replicates episomally and starts a new cycle (Fig. 1A) [33, 34].

HPV infections commonly induce benign hyperplasia, such as warts or papillomas, which usually resolve spontaneously. However, in rare instances, infected cells may undergo malignant transformation, often occurring a decade or more after the initial infection [35].

### Molecular drivers of cancer: HPV oncoproteins E6 and E7 in focus

HPV induces genetic, epigenetic, and metabolic changes that reprogram cellular processes and dysregulate key signaling pathways. These alterations allow cancerous cells to bypass cell cycle checkpoints, evade programmed cell death, and escape both innate and adaptive immune responses [36–38].

HPV oncogenes E6 and E7 are central to driving neoplastic transformation by disrupting the function of two key tumor suppressor proteins, p53 and RB.

The E6 protein promotes the degradation of p53 by facilitating its polyubiquitination through E6-associated protein (E6-AP) [39, 40]. E6 also impairs p53 transcriptional activity through several mechanisms: it interacts with CBP/p300 histone acetyltransferase [41, 42], inducing conformational changes that inhibit p53 function [43, 44], and retains p53 in the cytoplasm, preventing its nuclear translocation [45]. This degradation of p53 disrupts normal cell proliferation and induces resistance to apoptosis, activating cell cycle and anti-apoptotic pathways. E6 achieves this by inhibiting the transcription of cell cycle suppressor genes and pro-apoptotic genes or by repressing tumor suppressor miRNAs such as miR-23b, miR-218, and miR-34 [46].

By binding to MYC, E6 recruits E6-AP to the repressor NFX1-91, promoting its polyubiquitination and proteasome-mediated degradation. This degradation results in the transcriptional activation of telomerase reverse transcriptase (TERT), an enzyme responsible for maintaining telomere length and promoting cellular immortality [47–49]. By extending the lifespan of infected cells, this process supports the virus's long-term persistence.

Additionally, E6 disrupts cell differentiation by inhibiting the NOTCH1 signaling pathway. This occurs through E6 binding to NOTCH1 signal transducers such as MAML and SMAD2/3, which impair the normal differentiation process in infected cells [50–52]. E6 also promotes cell growth by degrading tuberous sclerosis complex 2 (TSC2) through the E6/E6-AP complex, leading to activation of the mTOR pathway, which stimulates anabolic processes that support increased cellular proliferation [53]. To meet the higher metabolic demands of these rapidly proliferating cells, E6 enhances glucose uptake by binding to and regulating sorting nexin 27 (SNX27). SNX27 is involved in retrograde transport from endosomes and in GLUT1 recycling to the cell surface, allowing greater nutrient uptake to sustain growth [54, 55].

Suppression of key cell cycle checkpoints, such as p53 and RB, results in uncontrolled cell proliferation, oxidative stress, and potential apoptosis, which could interfere with the virus's reproductive cycle. To prevent this, E6, in conjunction with E6-AP, blocks apoptosis by targeting the pro-apoptotic protein BAX for degradation [56–58]. In addition to promoting apoptosis resistance, E6 facilitates neoplastic transformation by degrading PDZ-domain proteins, such as PSD95/hDlg/ZO-1, which are crucial for maintaining cell polarity. E6 accomplishes this through its PDZ-binding motif (PBM) [59–61]. The degradation of PDZ-domain proteins leads to the loss of cell polarity, a hallmark of cancer cells that contributes to increased cell migration and metastasis (Fig. 1B).

In addition to E6, E7 is another key oncoprotein that plays a critical role in driving oncogenesis. While E6 primarily targets p53, E7 disrupts cell cycle regulation by targeting the tumor suppressor protein RB for proteasomal degradation. Following this degradation, the transcription factor E2F is released, which is essential for the transcription of genes involved in the cell cycle, such as cyclins A and E, promoting the transition from G1 to S phase [40, 62–66]. Furthermore, E7 interacts with E2F6, a component of the polycomb repressive complex (PRC), to derepress genes necessary for S-phase entry, allowing for uncontrolled DNA synthesis and cell cycle progression [67]. By ensuring that infected cells continuously progress through the cell cycle, E7 plays a central role in maintaining uncontrolled proliferation, thereby advancing the oncogenic process.

Beyond its role in disrupting cell cycle regulation, E7 also reprograms the gene transcription landscape of infected cells by modulating the activity of a diverse array of transcription factors, including STAT1, NF-κB, IRF1, SMAD2/3, TBP, ZMIZ1, MYBL2, MYC, JUN, and FOS [68]. A particularly critical aspect of E7 function is its ability to enhance cell proliferation by suppressing the expression of miR-203, a microRNA that normally acts as a repressor of the oncogene ΔNp63 [69] a key regulator of cell proliferation, stemness, and migration.

### Immune evasion: how E6 and E7 proteins escape host defenses

Immune evasion is crucial for HPV persistence, making modulation of host defenses key to viral survival. In this process, E6 and E7 work together, effectively reprogramming the immune response to ensure the virus can evade detection.

E6 impairs the innate immune response by blocking the transcription of IFN-β, a key antiviral signaling molecule, through its binding to the interferon regulatory factor 3 (IRF-3) transcription factor [70]. Additionally, E6 inhibits the IFN-α-mediated response by preventing the autophosphorylation of Tyk2 and ISG3α, two essential components of the ISGF3 transcription factor complex. This suppression of the interferon response cripples the cell's ability to mount an effective antiviral defense [70]. Through E6-AP–mediated ubiquitination, E6 also degrades IL-1β, suppressing innate defenses [36] (Fig. 1B).

E7 binds to IRF-1 and IRF-9 (key interferon regulators) thereby suppressing innate signaling [71, 72]. Furthermore, E7 interferes with the cGAS-STING pathway, a key sensor of intracellular viral DNA that typically triggers an innate immune response. By interacting with STING, E7 prevents activation of the immune response, further aiding the virus in evading the host’s defenses (Fig. 1C) [73, 74].

E7, alongside E6, suppresses the expression of Toll-like receptor 9 (TLR9), which serves as the primary sensor of intracellular double-stranded DNA (dsDNA). By downregulating TLR9, E7 impairs the host’s ability to recognize viral DNA, further enabling immune evasion [75]. E7 binds TNFR1 to block TNFα-mediated signaling, dampening inflammation and impairing clearance of infected cells. E7 also represses the expression of TGFβ, a cytokine critical for immune regulation and inflammation, allowing the virus to further evade immune surveillance [24].

### Creating chaos: the role of HPV in inducing genomic instability and epigenetic reprogramming

In addition to immune evasion, E7 disrupts centrosome duplication, driving chromosomal instability. In concert with E6, this disruption leads to mitotic errors and chromosomal instability, both of which are key drivers of tumor development. The oncoprotein E7 induces abnormal centrosome duplication through the downregulation of RB, which functions as an inhibitor of cell cycle progression by suppressing the activity of E2F transcription factors. By inactivating RB, E7 unleashes E2F, prematurely activating cyclin E/CDK2 and decoupling centrosome duplication from mitosis [76–78]. E7 also delocalizes nuclear mitotic apparatus protein 1 (NuMA), impairing spindle pole organization [79]. It further reduces γ-tubulin recruitment by disrupting dynein-mediated transport [80, 81]. Complementing these actions, E6 exacerbates centrosome abnormalities by upregulating PLK1, a key regulator of centrosome assembly, through p53 degradation, further promoting mitotic errors and centrosome dysfunction (Fig. 1D) [82]. Additionally, E6 contributes to genomic instability by causing the accumulation of mutations in the host genome through its binding to the DNA repair protein XRCC1, impairing its function [83]. These mutations further drive the transformation of infected cells into cancerous ones.

The expression of E6 and E7 oncoproteins also induces significant epigenetic reprogramming in the host cell genomic DNA, contributing to tumorigenesis. E6 through degradation of p53 via the E6/E6-AP complex, and E7 through its downregulation of RB lead to the release of SP1 and E2F transcription factors, respectively. This release activates the transcription of DNA methyltransferase 1 (DNMT1), which results in the hypermethylation of promoter regions and subsequent silencing of tumor suppressor genes, such as APC1 [84]. This silencing plays a critical role in allowing uncontrolled cell growth. Furthermore, the E6/E6-AP mediated degradation of the histone H3K4 demethylase KDM5C further alters the epigenetic landscape by promoting the expression of genes associated with cell proliferation [85] (Fig. 1D).

While the expression of E6 and E7 oncoproteins affects a wide array of signaling pathways and induces significant genomic, epigenetic, and metabolic alterations, including the downregulation of tumor suppressor genes, inhibition of DNA repair, centrosome dysregulation, evasion of cell cycle checkpoints, and immune suppression, these changes alone are not sufficient for full tumorigenesis [77, 83, 86–88]. A crucial step in the neoplastic transformation of CSCC and OPSCC is the integration of viral DNA into the host genomic DNA. This integration of viral DNA effectively ensures the continuous disruption of key regulatory pathways, driving to the progression of pre-malignant cells into invasive tumors. This critical transition from an episomal to an integrated state thus marks a turning point in HPV-induced oncogenesis, shifting the focus from viral survival to host cell transformation and cancer development.

### HPV DNA integration: the turning point from infection to malignancy

Although HPV infection is common, progression to malignancy is rare and typically occurs ≥ 10 years post-infection. Risk factors for persistent infection and progression include: immunocompromised status, unhealthy behaviors like smoking and excessive alcohol consumption, the presence of other sexually transmitted infections (such as HIV), poor hygiene, and alterations in the local microbiota [24, 89–92].

A critical factor in HPV-associated cancer development is the integration of viral DNA into the host genome, which occurs in 70–85% of HPV-positive cancers [93]. This integration locks infected cells into a state of persistent oncogenic signaling, pushing them toward malignant transformation.

Integration aborts the viral life cycle (no new virions are formed) shifting the virus to a latent, non-replicative state. The absence of viral replication reduces immune detection, while the continued expression of E6 and E7 oncoproteins accelerates tumor progression by silencing tumor suppressors and promoting genomic instability [94–96].

Viral integration occurs in vulnerable regions of the host genome called fragile sites, allowing HPV to establish a persistent presence within the host cell and immortalize it for long-term survival. Fragile sites are regions prone to chromosomal breakage during mitosis, particularly under replication stress conditions, such as DNA secondary structure formation, inadequate replication origin firing, or replication-transcription interference. These factors can lead to fork collapse and DNA breakage, providing entry points for viral DNA integration [94–96]. These fragile sites are specific to certain cell types and are influenced by the epigenetic landscape and chromatin conformation, which respond to stimulus-induced gene expression in the tissue environment [97]. E6/E7-driven hyperproliferation depletes nucleotide pools, stalling replication forks. Although MCM helicase persists, single-stranded DNA accumulates, activating ATR and TLS via POLη [98–100]. HPV proteins then impair TLS, leading to fork collapse and DSB formation.

By integrating into the host DNA, the virus often disrupts key regulatory regions, such as the E2 ORF. This disruption is significant because E2 normally represses the expression of E6 and E7. Without this control, E6 and E7 become overexpressed, driving uncontrolled cell proliferation [40, 101, 102].

In addition to removing this regulatory check, viral integration further enhances the virus ability to persist within the host. Viral–host fusion transcripts from integrated HPV DNA are more stable than episomal viral RNAs, ensuring sustained E6/E7 expression [103].

Integration may generate tandem repeats that function as super-enhancers, further amplifying E6/E7 transcription and oncogenic signaling (Fig. 1A) [104].

Since E6 and E7 expression levels are low in some HPV-positive tumors, there must be alternative mechanisms driving tumor development [105]. Viral DNA integration into the host genome can disrupt critical regions responsible for the regulation of oncogenes and tumor suppressor genes. Disruption occurs through alterations in the ORFs and modifications to promoter activity, which can significantly alter gene expression [93]. Viral integration can also lead to inactivation of the *RAD51B* gene, which is essential for DNA repair via homologous recombination (HR). When *RAD51B* is inactivated, the cell ability to repair DSBs is impaired, promoting genomic instability and an increased risk of cancer progression [106, 107]. Additionally, intrachromosomal rearrangements caused by viral integration can result in the upregulation of oncogenes or the downregulation of tumor suppressor genes, further destabilizing the genome. These rearrangements can also introduce changes in epigenetic markers, such as DNA methylation, which can silence tumor suppressor genes near the viral integration site, further contributing to tumor development [107, 108].

### DNA damage and repair: navigating the double-edged sword of HPV-driven cancers

Once DSBs and single-stranded breaks (SSBs) occur, these disruptions enable the integration of viral DNA into the host genome. Although this may appear to be a setback for the viral life cycle, it facilitates viral replication by attracting DNA repair factors and HR proteins to viral replication centers, where viral integration takes place [29].

Unrepaired DSBs/SSBs cause chromosomal abnormalities that are detrimental to the host cell. To counter this, cells activate two primary DNA repair pathways: HR and non-homologous end-joining (NHEJ) [109, 110]. NHEJ can operate throughout the cell cycle and quickly repairs DNA breaks by directly ligating the broken ends. However, this pathway is error-prone because it doesn’t use a template for repair, which can lead to mutations or chromosomal rearrangements. In contrast, HR is an error-free repair mechanism that functions only during the S/G2 phase of the cell cycle. This pathway requires a template, typically a sister chromatid, to guide the repair of the DSB, ensuring that the repaired DNA is accurate and stable [111, 112].

The HR pathway is initiated when the MRN complex, consisting of MRE11, RAD50, and NBS1, detects the free ends of a DSB and recruits ATM to the damage site [113]. ATM is a kinase that plays a pivotal role in the cellular DDR. Once activated, ATM phosphorylates a range of proteins critical to the repair process, including PALB2, RAD51, and histone H2AX. The phosphorylation of H2AX marks the chromatin surrounding the DSBs and acts as a signal to recruit other repair proteins. The RAD51 recombinase is especially important because it facilitates the formation of nucleoprotein filaments, which are essential for homology searching and strand invasion during DNA repair [114]. Next, proteins such as MRE11, CtIP, DNA2, and EXO1 initiate the resection of the broken DNA ends, creating ssDNA overhangs. This ssDNA is crucial because it allows for the homology search, where the damaged DNA is paired with an intact template from a sister chromatid, ensuring error-free repair [114].

Once ssDNA is generated, it is rapidly coated by RPA, which stabilizes the ssDNA and prevents the formation of harmful secondary structures. RPA also acts as a platform for the recruitment of ATR and its binding partner ATRIP, driving the activation of a DNA damage checkpoint. This checkpoint ensures that the cell cycle is paused to allow sufficient time for the repair of the damaged DNA [115, 116]. Specifically, ATR phosphorylates CHK1 which further regulates the cell cycle by halting progression until the DNA is adequately repaired. ATR also phosphorylates several other key proteins, including RPA32, SMC1, and RAD9, all of which are involved in coordinating repair processes [117, 118]. Following checkpoint activation, the ATM-regulated BRCA1-BRCA2-PALB2 complex is recruited to the damaged DNA and helps load RAD51 recombinase which removes RIF1 by generating helical nucleoprotein filaments that scan genomic DNA for a complementary sequence [119–121]. When the correct sequence is found, the RAD51-coated strand invades duplex recipient DNA, forming a displacement loop (D-loop) that can be processed by three major pathways: synthesis-dependent strand annealing (SDSA), break-induced replication (BIR), and DSB repair (DSBR) [122–125].

In the DSBR pathway, after D-loop formation, DNA polymerases synthesize the complementary strand using the 3’-OH group exposed post-resection, with the sister chromatid as a template for accurate repair. Ligases then join these newly synthesized strands, forming a double-Holliday junction (HJ), which are critical intermediates in DNA recombination. Proper resolution of HJs, mediated by the STR complex (including BLM, TOPOIIIα, RMI1, and RMI2) and specific nucleases, ensures DNA stability and can result in either crossover or non-crossover outcomes [126–128]. In the SDSA pathway, the newly synthesized DNA detaches from the homologous template and reanneals with other 3’ overhangs on the damaged chromatid. DNA polymerase fills gaps, forming non-crossover recombinant DNA, thus avoiding genetic exchange between chromosomes and ensuring conservative repair [129–131]. BIR addresses extensive DNA damage, particularly when the damages extend to the replication fork or chromosome end. The invading strand attaches to a homologous DNA molecule, continuing DNA synthesis until the next replication fork or the chromosome end. Polδ, Polε, and Polα utilize the newly synthesized guide DNA as a template for lagging strand synthesis, forming Okazaki fragments to finalize replication [122, 132–134]. Notably, most DSB repair during mitosis occurs via the SDSA pathway to minimize crossover and genomic instability.

Despite NHEJ's lower precision compared to HR, its rapidity is crucial for genomic stability in non-dividing cells. The NHEJ pathway initiates when the Ku heterodimer (Ku70 and Ku80) detects a DNA DSB and binds to the broken ends. Ku recruits and activates DNA-dependent protein kinase catalytic subunit (DNA-PKcs), the central NHEJ assembly hub [135, 136]. Activated DNA-PKcs phosphorylates several repair proteins, coordinating the pathway. The Ku/DNA-PKcs complex then recruits XRCC4, XLF, and PAXX, which stabilize the DNA ends, facilitating the assembly of the repair machinery. Enzymes like PNKP, Apratxin, Artemis, APLF, and WRN process the damaged DNA [137–146]. DNA polymerases μ and λ fill gaps, and the XRCC4/DNA ligase IV complex ligates the DNA ends, completing the repair [147, 148]. While efficient, NHEJ often introduces mutations or small deletions due to the absence of a homologous template.

Competition between HR and NHEJ for DSB repair is primarily regulated by the DDR pathway, controlled by the ATM and ATR kinases. These kinases ensure that HR is chosen over NHEJ when error-free repair is feasible. HR is promoted by the MRE11/CtIP complex and regulated by CtIP phosphorylation via ATM and CDK. Phosphorylation enhances the activity of MRE11/CtIP complex, initiating DSB end resection to create a single-stranded DNA overhang necessary for HR [110, 149]. Concurrently, BRCA1 binds to CtIP and the MRN complex, further stimulating resection and preventing the Ku complex from binding and initiating NHEJ [150]. BRCA1 also inhibits NHEJ by inducing 53BP1 dephosphorylation, leading to RIF1 release. This prevents RIF1 accumulation at the DSB site, blocking NHEJ and ensuring that HR proceeds [149, 151]. These mechanisms prioritize HR during the S/G2 phase to maintain genome stability while maintaining NHEJ as a secondary option for non-dividing cells.

E6/E7 reduce HR efficiency by approximately 50–60%, accelerating genomic instability. HR impairment is primarily mediated through E7-induced downregulation of TGFβ, which subsequently derepresses miR-182, promoting HR inhibition and error-prone repair mechanisms. miR-182 downregulates BRCA1 and FOXO3, which are critical factors in DNA repair, thereby impairing the ATM activation, which is necessary for HR [152, 153]. The E6 protein further compromises HR through two mechanisms: first, by facilitating the degradation of TIP60, a crucial acetyltransferase for ATM activation, which consequently hinders proper ATM activation and diminishes HR initiation [154, 155]; second, by causing the displacement of RAD51 from DNA damage foci, ultimately contributing to genomic instability [156]. Additionally, E6 initiates HR during the G1 phase, which is harmful because HR normally occurs in the S/G2 phase, where sister chromatids serve as repair templates. E7 disrupts HR by interfering with BRG1 ATPase, which promotes DNA end resection by reducing nucleosome density around DSBs [157]. This reduction in nucleosome density is necessary for HR to proceed effectively, as it allows access to repair proteins. E7 also hijacks RNF168, an E3 ubiquitin ligase that ubiquitinates histone H2A at DSBs. The ubiquitination of H2A is essential for recruiting the 53BP1/RIF1/shieldin complex, which inhibits DNA resection and promotes NHEJ. The E7-RNF168 interaction can direct the host ubiquitin machinery to the viral chromatin, ensuring the recruitment of HR factors to the viral DNA for rapid repair and amplification. RNF168 hijacking by E7 away from DSBs impairs DNA repair by altering histone H2A ubiquitination and interfering with 53BP1 recruitment (Table 1) [158–162].

### HPV DNA integration as a catalyst for malignancy

HPV DNA integration into the host genome significantly increases cancer risk, driving progression from precancerous to malignant lesions. This integration step is a key event that drives tumorigenesis by altering cellular regulatory mechanisms. Viral DNA integrates into either intronic or exonic regions, each with distinct cellular consequences. Integration into introns disrupts chromatin structure and regulatory functions. In exons, it can reduce gene expression, create truncated proteins, or drive oncogene amplification. The consequences of HPV integration are not uniform and can vary depending on the affected genes. For example, HPV integration has been shown to reduce the expression of tumor suppressor genes such as *FHIT* and *LRP1B*, while simultaneously promoting the expression of oncogenes like *MYC* and *HMGA2* [105, 163, 164]. These disruptions in gene regulation provide a selective advantage for the cells, pushing them further toward malignancy.

Although both CSCC and OPSCC rely on HPV integration, the affected genes differ, leading to distinct behaviors and clinical courses. For example, in HPV-associated CSCC, recurrent integration regions include *POU5F1B, FHIT, KLF12, KLF5, LRP1B, LEPREL1, HMGA2, DLG2*, and *SEMA3D* [163, 164]. Common integration sites in HPV-positive OPSCC include genes like *RAD51B, MACROD2, NR4A2, KLF5, KLF12, MYC*, and *TP63,* some of which are also found in CSCC. Additionally, the 9p24.1 region, containing genes such as *PDL1, PDL2*, and *PLGRKT*, is frequently integrated in OPSCC. These genes are involved in immune checkpoint pathways, playing an immunosuppressive role that allows tumor cells to evade the immune system [105, 165, 166].

Gene-expression profiling divides HPV-positive CSCC and OPSCC into two subtypes, defined by integration patterns and downstream transcriptional changes. The Immune subtype, lacking integration, shows strong immune signatures and better prognosis. The Keratinization subtype, with integrated HPV, exhibits keratin-related gene expression, active WNT/β-catenin signaling, and poorer outcomes (Table 2) [167–172].

### Diverging pathways: comparing HPV-positive cervical and oropharyngeal squamous cell cancers

HPV-positive OPSCC and CSCC share biology but differ markedly in natural history and treatment response compared to HPV-negative tumors. HPV employs distinct mechanisms in each tissue to promote tumorigenesis, influenced by a variety of factors, including tissue origin, hormones, growth factors, tissue-specific cytokines, immune system composition, chromatin conformation, epigenetic features, HPV variants, E6 viral gene mutations, and viral DNA integration sites. These differences in tumorigenic mechanisms help explain the varying susceptibilities of HPV-positive cancers to different treatments and highlight the importance of tailoring investigations and therapies for each specific cancer type.

**Fig. 2:**
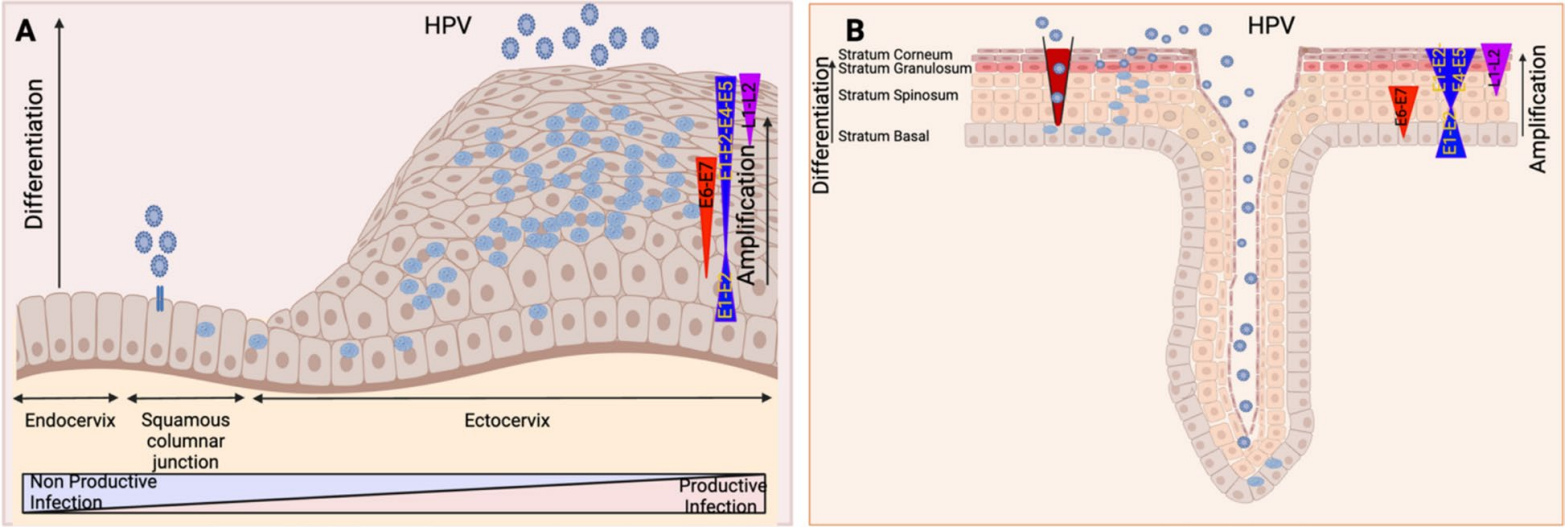
HPV infection in the cervix and tonsil crypts. **A)** The cervix includes three epithelial types: stratified squamous epithelium (external cervix), monostratified columnar epithelium (internal cervix), and the transformation zone (TZ) between them. The squamocolumnar junction (SCJ) marks the boundary between squamous and columnar epithelium. HPV can infect basal layer cells via microabrasion or SCJ cells. In the TZ, improper regulation of viral gene expression leads to non-productive infections, unlike productive infections observed in the ectocervix. Epithelial differentiation triggers the PE promoter to drive E6 and E7 gene expression for S-phase entry (red). The PL promoter becomes upregulated in the upper epithelial layers, increasing viral replication protein levels (E1, E2, E4, E5) (blue) for genome amplification. In the upper layers, late L1 and L2 genes are expressed to encapsulate viral genomes, forming progeny virions in the nucleus, which can initiate new infections. **B)** The oral epithelium consists of basal, spinous, granular, and corneous layers. In contrast, tonsil crypts have a reticulate epithelium, less organized than stratified squamous epithelium, containing epithelial and non-epithelial cells, particularly lymphoid cells essential for immune function. This thin epithelial layer sometimes lacks a basement membrane, improving antigen capture and immune surveillance but increasing viral infection risk. HPV infects the basal layer of the stratified epithelium via microabrasion but can easily access the tonsils basal cells. Inside, the virus amplifies transiently to 50–100 copies per cell, maintaining stable numbers in undifferentiated basal cells with cellular DNA replication. Differentiation activates the productive phase, leading to late gene expression and genome amplification to thousands of copies per cell. E6 and E7 enable cell cycle re-entry post-differentiation for productive replication, with E4 and E5 contributions. L1 and L2 expression encapsulates the replicated genomes, resulting in virion release from the upper epithelium layers (created with BioRender.com)

CSCC typically arises in the transformation zone, located between the squamous epithelium of the ectocervix and the columnar epithelium of the endocervix [173]. This region is maintained by a population of stem-like cells known as reserve cells, which play a key role in tissue integrity and regeneration [174]. However, these reserve cells are also believed to be the primary source of CSCC. Due to their stem-like properties, reserve cells have the ability to alter viral gene expression or promote HPV integration into the host genome [175]. By preventing HPV from completing its full replication cycle, this integration, leads to an abortive infection that significantly increases the risk of neoplastic transformation (Fig. 2A).

In contrast, OPSCC primarily originates from the reticular epithelium of the tonsillar crypts, which are rich in epithelial progenitor cells expressing stem cell markers such as CD44 and NGFR [176, 177]. Similar to CSCC, HPV infects these progenitor cells, disrupting the viral replication cycle and driving an abortive/non-productive infection that promotes malignant cell transformation [178, 179]. These HPV-infected progenitor cells can further evolve into cancer stem cells, expressing markers such as CD44 and ALDH1, which are associated with self-renewal, invasion, and metastasis (Fig. 2B) [180–182].

Despite the commonality that both reserve cells in the cervix and epithelial progenitor cells in the oropharynx act as targets for HPV infection, these cells play different biological roles and show varying proliferation rates. Furthermore, while infected OPSCC progenitor cells consistently express CD44 and ALDH1, infected cervical reserve cells show variable expression levels of these markers, preventing their definitive identification. Consequently, while OPSCC progenitor cells can be easily identified and targeted through therapies specific to CD44 and ALDH1, this is not applicable to cervical cancer stem cells, thus limiting therapeutic possibilities. This highlights the complexity of HPV-driven tumorigenesis and the need for disease-specific therapeutic strategies.

Although HPV16 is the most commonly detected genotype in both CSCC and OPSCC, there are notable differences in the distribution of other HPV genotypes between the two cancer types. HPV18 and HPV45 are more prevalent in CSCC, whereas HPV35 is found more frequently in OPSCC [183]. Geographic differences in HPV variants also affect the risk of developing these tumors, with European and Asian variants being more common in OPSCC than in CSCC [184]. In addition to the differences in HPV genotypes, the mutation rate of the E6 oncogene varies significantly between these cancers. In OPSCC, E6 mutations occur in 18.5% of tumors, whereas in CSCC, the rate is much lower, at 2.0%. Interestingly, no significant mutational differences were found in the E7 oncogene between these tumor types, which suggests that the E7 gene plays a highly conserved role in HPV-driven oncogenesis [183]. The higher E6 mutation rate in OPSCC may reflect selective pressures in the oropharyngeal environment, where the virus adapts to different physiological conditions.

HPV-positive CSCC and OPSCC share certain mutational patterns while also exhibiting unique ones. The overlapping and divergent pathways indicate that while HPV drives tumorigenesis through common molecular mechanisms in CSCC and OPSCC, the specific tissue environment and genetic context influence the trajectory of the disease.

Common mutations: Certain genes, including *PIK3CA, PTEN, TP53, KRAS, EP300, FBXW7, and HLA-A/B*, are frequently mutated in both CSCC and OPSCC tumors. These genes are involved in key cellular processes such as cell growth, survival, and immune response, highlighting their role in HPV-driven carcinogenesis across both cancer types [164, 185, 186].

The mutated genes in both tumor types fall within several key biological pathways:

- PI3K/AKT, RAS/EGFR/ERK, TGF-β, RB, and MEK/ERK pathways are critical for cell growth and survival.
- NF-kB and MHC pathways are integral to immune responses.
- NOTCH and DNA repair pathways regulate cell differentiation and genomic integrity [185, 186].

Unique mutations: However, specific mutations differentiate the two cancers. In CSCC, mutations are more often found in genes such as *EGFR, SMAD4, ERBB2, ERBB3, ELF3, TGFBR2, CREBBP, MAPK1c, CBFB, ARID1A, NFE2L2, CASP8, STK11, SHKBP1, LKB1*, and *NOL7*. These genes are involved in pathways regulating cell signaling, growth, and differentiation [185, 186].

Conversely, in OPSCC, mutations are more frequently found in genes such as *RB1, FGFR2, FGFR3, MLL2, MLL3, ASXL1, NOTCH1, ATM, BRCA1, NF1, FLG, BRCA2, LRP1B, HRAS, TRAF3, DDX3X, TPRX1, CYLD, RIPK4*, and *UBR5* [164, 185, 186]. These genes are more directly involved in chromatin remodeling, immune regulation, and DNA repair mechanisms, underscoring the importance of genomic stability in OPSCC progression.

The mutations found in these genes are attributed to the activity of the apolipoprotein B mRNA editing enzyme, catalytic polypeptide-like (APOBEC) family of cytosine deaminases. They normally function as part of the innate immune response editing viral genomes and inducing mutations to reduce viral infectivity. This mechanism is an essential part of the body`s defense against viral infections, including HPV. However, in the context of HPV infection, this protective response becomes a double-edged sword. HPV infection and the expression of the E6 oncoprotein lead to the upregulation of APOBEC enzymes. Although APOBEC activity can reduce HPV infectivity by introducing mutations in the viral genome, it simultaneously creates unintended genomic instability within the host, driving the accumulation of mutations in key oncogenes and tumor suppressor genes, contributing to cancer progression [107, 187]. Since they induce cytosine-to-uracil conversions, resulting in C-to-T transitions, APOBEC-induced mutations are characterized by a specific mutational signature in both CSCC and OPSCC tumors. In both CSCC and OPSCC, these mutations significantly contribute to the cancer's mutational burden, influencing its aggressiveness and the response to therapy.

### Chromosomal alterations in HPV-positive tumors

HPV-positive tumors also display structural DNA alterations, oncogene amplifications and tumor-suppressor deletions, that drive tumorigenesis. For both cancer types, the 3q region is notably affected, with amplification of the mutated *PIK3CA* gene observed in both CSCC and OPSCC tumors [186, 188]. However, the specific amplified or deleted chromosomal regions vary between the two cancers, reflecting the distinct mechanisms of tumorigenesis.

CSCC-specific amplifications include 3q28 (harboring TP63 and LPP), 3q24.1 (TGFBR2), 18q21.2 (SMAD4), and 7p11.2 (EGFR); frequent deletions occur at 3p14.1 (FOXP1) [186, 188].

In contrast, the most commonly amplified regions in OPSCC are 3q26.33, which contains the transcription factor *SOX2* involved in self-renewal [186, 189]; 3q27.1, which includes the *KLHL6* gene that regulates immune signaling; and 3q27.3, containing the *BCL6* gene, a key player in the RTK-JAK-STAT signaling pathway [190]. Other OPSCC amplifications include 5p13.1, harboring *RICTOR*, a component of the PI3K/AKT pathway; 8q24.21, which contains the *MYC* oncogene; 11q13.3, which contains *FGF19*, *FGF3*, and *FGF4*, all involved in RAS/EGFR/ERK pathway regulation; and 14q32.33, which contains the *AKT1* oncogene. On the other hand, key regions frequently lost in OPSCC include 4q31.3, which harbors the *FBXW7* gene involved in Notch signaling; 13q14.2, which contains the tumor suppressor gene *RB1*; 14q32.32, which includes *TRAF3*, involved in immune responses; and Xp11.3, harboring *KDM6A*, a histone demethylase involved in chromatin organization [186, 190].

PIK3CA amplification at 3q is shared, underscoring PI3K/AKT pathway activation. However, CSCC shows focal-adhesion and growth-factor signaling amplifications (*TP63, TGFBR2, LPP*), whereas OPSCC is characterized by self-renewal and immune-signaling amplifications (*SOX2, BCL6, MYC*) and deletions (*FBXW7, RB1, TRAF3*).

### Divergent radiosensitivity: clinical implications and challenges

HPV status differentially shapes radiotherapeutic management in OPSCC compared to CSCC. In OPSCC, robust evidence confirms that HPV-positive tumors respond more favorably to radiation than their HPV-negative counterparts, allowing for de-intensified protocols that maintain high survival rates. For instance, Almangush et al. (2022) [191] reported no decrease in survival when the total dose was reduced from 70 to 60 Gy. Similarly, the phase II Optima trial [192] and the phase III Quarterback trial [193] observed two-year progression-free survival (PFS) equivalent to standard doses when patients received 45–56 Gy after induction chemotherapy, and the NRG HN02 study found comparable PFS (90.5% vs. 87.6%) with 60 Gy instead of the full 70 Gy [194]. Notably, overall survival remained similar between de-escalated and conventional arms. ASTRO’s 2024 guidelines [195] now recommend lower radiation doses in HPV-positive OPSCC, reflecting these deintensification data. Beyond oncologic outcomes, such de-intensification eases treatment-related burdens, lowering the incidence of severe dysphagia, mucositis, and xerostomia [196], and ultimately enhancing the quality of life. This evolution is further codified in the 8th edition of the UICC/AJCC TNM staging, where HPV-positive OPSCC is assigned more favorable categories [11]. By contrast, HPV status does not currently alter the treatment strategy for CSCC. Despite HPV16 and HPV18 being implicated in approximately 70% of these tumors [197–199], no radiosensitivity advantage over HPV-negative CSCC has been conclusively demonstrated. Given the rarity of truly HPV-negative CSCC, it remains unclear whether sample-size limitations mask a genuine biological difference. Furthermore, as HPV vaccination programs advance, they will likely shift the epidemiology of cervical cancer, potentially enabling larger comparative analyses of the rare HPV-negative subset in the future. As a result, both HPV-positive and HPV-negative CSCC follow the same aggressive approach of concurrent chemoradiotherapy plus intracavitary brachytherapy (BT), with recommended total doses reaching 80–90 Gy [12]. This regimen ensures adequate tumor coverage and local control but carries a higher toxicity burden, including gastrointestinal, genitourinary, and hematologic adverse events, which can be exacerbated by extended-field radiation [200, 201]. Although brachytherapy remains essential for dose escalation directly at the tumor site, either intracavitary if tumor reduction exceeds 50% or interstitial otherwise [202], no dose de-escalation trials exist to parallel those in OPSCC. In cases where brachytherapy is infeasible, stereotactic body radiation therapy offers an alternate route to achieve a similar bioequivalent dose [203]. Hence, while HPV positivity has driven meaningful changes in OPSCC management, it has yet to alter CSCC protocols. Future epidemiological shifts, particularly from vaccination programs, may, clarify whether a subset of HPV-negative CSCC emerges with distinctly different sensitivities, prompting new therapeutic paradigms if larger comparative data become available.

## Conclusions

The divergent radiosensitivity of HPV-positive OPSCC versus CSCC underscores the need to elucidate the underlying molecular mechanisms. While both cancers share common tumorigenic pathways, including the inactivation of p53 and pRB tumor suppressor proteins by HPV E6 and E7 oncoproteins [204, 205], these disruptions result in increased radiosensitivity in OPSCC but not in CSCC. Understanding the biological underpinnings of these divergent responses is crucial to optimizing therapeutic outcomes and reducing treatment-related toxicity.

Several potential explanations may account for this clinical discrepancy. One possibility is that, while HPV infection is a necessary factor in both cancers, it may not be sufficient on its own to explain their differing radiosensitivity. Although HPV infection is necessary, additional genetic, epigenetic, hormonal, and immune factors likely modulate radiation response in each site [206–208]. These factors could modulate the way host cells respond to radiation therapy, influencing radiosensitivity in distinct ways between the two cancers.

Nevertheless, caution is required when translating biological insights directly into clinical practice. Although certain de-intensification trials in HPV-positive OPSCC have demonstrated comparable survival rates, other studies challenge these findings in advanced disease stages or among heavy smokers, often citing short follow-up intervals and limited applicability of therapy de-escalation in these subgroups [11]. Furthermore, relying solely on p16 immunohistochemistry to define HPV status has recognized limitations in specificity, as p16 overexpression does not always equate to bona fide HPV-driven carcinogenesis. Some patients exhibit discordant results between p16 immunohistochemistry and HPV DNA or mRNA testing, raising legitimate concerns about single-method diagnostic approaches [11]. Additional nuances arise from the 8th edition of the TNM staging system, which, while an improvement over its predecessor, has been criticized for weak discrimination between certain stages, particularly stages II and III in HPV-positive patients, where survival outcomes are insufficiently distinct. Moreover, inconsistencies between clinical and pathological staging (the former relies on radiological findings, the latter on nodal metastases) further complicate accurate prognosis and treatment guidance [11]. The validity of survival estimates for specific staging groups, such as pathological stage III, may also be compromised by the relatively small number of patients included in the ICON-S study on which these criteria are based [11]. In light of these considerations, current guidelines recommend de-escalation primarily for carefully defined favorable-risk subgroups, underscoring the critical need for robust, long-term clinical data and multimodal diagnostic assessments to confirm or refute hypotheses derived from emerging biological insights [11]. The clinical relevance of HPV integration into the host genome is multifaceted. Specific regions of viral DNA integration can help guide prognosis and treatment strategies. For instance, patients with immune subtype HPV-driven tumors, which often lack HPV integration, have a more favorable prognosis and might benefit from immune-based therapies like checkpoint inhibitors [105, 165]. In contrast, tumors with HPV integration, commonly falling under the keratinization subtype, are linked to lower immune activity and poorer outcomes, necessitating more aggressive treatment approaches that combine viral oncogene targeting and immune checkpoint inhibition [166].

Additionally, viral integration disrupts key tumor suppressor genes and enhances immune evasion, particularly through the PDL1/PDL2 pathway, prevalent in HPV-positive OPSCC [164]. This presents a therapeutic opportunity for immune checkpoint inhibitors like anti-PD1 and anti-PDL1 therapies, offering significant benefits in tumors utilizing these pathways for immune escape [209]. Molecular markers of HPV integration, such as immune response profiles or gene amplifications like *MYC* and *HMGA2*, are poised to improve patient stratification for tailored treatments [107]. Further investigation is required to explore the influence of genetic and epigenetic variations on the abscopal effect of radiotherapy in advanced and metastatic CSCC and OPSCC. This phenomenon, characterized by tumor regression at both irradiated primary sites and distant metastases beyond the radiation field, is driven by systemic immune activation. Consequently, establishing the relationship between the antitumor response to radiotherapy and immunotherapy, with consequent metastasis eradication, and the genetic and epigenetic profiles of CSCC and OPSCC could provide valuable insights into the efficacy of this therapeutic approach for aggressive malignancies, potentially shaping future treatment strategies.

Another plausible explanation lies in the available epidemiological data. Unlike OPSCC, where significant differences between HPV-positive and HPV-negative cases have been extensively studied, HPV-negative CSCC has a lower prevalence compared to HPV-positive OPSCC. This difference may have limited the depth of analysis in the CSCC studies, potentially obscuring any significant differences in radiosensitivity. Current studies may not have been large enough or sufficiently powered to detect differences in radiosensitivity between HPV-positive and HPV-negative CSCC. This suggests the need for larger epidemiological studies and more comprehensive datasets to fully understand whether a true radiosensitivity difference exists or whether current data have not captured potential nuances in treatment response.

While high‐coverage HPV vaccination remains the cornerstone of cancer prevention, driving incidence in some countries to near‐zero and starkly contrasting with regions lacking robust programs [210], advances in molecular profiling are poised to transform diagnostics and treatment. High-throughput assays for HPV integration will enhance risk stratification, guide personalized therapeutic choices, and refine radiosensitivity predictions. By marrying population-level vaccine successes with individualized molecular insights, we can simultaneously prevent new infections and deliver tailored, less toxic treatments for HPV-driven malignancies, maximizing patient benefit and minimizing treatment burdens.

## Data Availability

Data sharing is not applicable to this article, as no new datasets were generated or analyzed during the current study.

## Abbreviations

AJCC: American Joint Committee on Cancer
ALDH1: Aldehyde Dehydrogenase 1
APOBEC: Apolipoprotein B mRNA Editing enzyme, Catalytic polypeptide-like
APC1: Adenomatous Polyposis Coli 1
APLF: Aprataxin And PNKP Like Factor
ARID1A: AT-Rich Interaction Domain 1A
ASXL: ASXL Transcriptional Regulator
ATM: Ataxia Telangiectasia Mutated
ATR: Ataxia Telangiectasia and Rad3-related
ATRIP: ATR Interacting Protein
BCL6: BCL6 Transcription Repressor
BIR: Break-induced Replication
BRCA1: BRCA1 DNA Repair Associated
BRD4: Bromodomain Containing 4
BRG1: Brahma‐related gene 1
BT: Brachytherapy
CBFB: Core Binding Factor Beta
CBP/p300: CREB-binding protein/p300
CD44CD63, CD151: Cluster of Differentiation 44, 63, 151
CDK: Cyclin-dependent Kinase
CDKN2A (p16): Cyclin-dependent kinase inhibitor 2A
cGAS: Cyclic GMP-AMP Synthase
CHK1: Checkpoint Kinase 1
CREBBP: CREB Binding Protein
CSCC: Cervical Squamous Cell Carcinoma
CtIP: CtBP-interacting Protein
CYLD: CYLD Lysine 63 Deubiquitinase
DDR: DNA Damage Response
DDX11: DEAD/H-Box Helicase 11
DDX3X: DEAD-Box Helicase 3 X-Linked
DLG2: Discs Large MAGUK Scaffold Protein 2
DNA-PKcs: DNA-dependent protein kinase catalytic subunit
DNMT1: DNA Methyltransferase 1
DSB: Double-Stranded DNA Break
DSBR: Double-Stranded Break Repair
E2F6: E2F Transcription Factor 6
EBV: Epstein-Barr Virus
EGFR: Epidermal Growth Factor Receptor
ELF3: E74-like Factor 3
EP300: E1A Binding Protein P300
ERBB2, ERBB3: Erb-B2 Receptor Tyrosine Kinase 2, Erb-B2 Receptor Tyrosine Kinase 3
EXO1: Exonuclease 1
FBXW7: F-box and WD Repeat Domain Containing 7
FGF3, 4, 19: Fibroblast Growth Factor 3, 4, 19
FGFR2, FGFR3: Fibroblast Growth Factor Receptor 2, Fibroblast Growth Factor Receptor 3
FHIT: Fragile Histidine Triad
FLG: Filaggrin
FOS: Fos Proto-Oncogene, AP-1 Transcription Factor Subunit
FOXO3: Forkhead Box O3
FOXP1: Forkhead Box P1
GLUT1: Glucose Transporter 1
Gy: Gray (unit of radiation dose)
hDlg: Discs Large MAGUK Scaffold Protein
HLA: Human Leukocyte Antigen
HMGA2: High Mobility Group AT-hook 2
HPV: Human Papillomavirus
HR: Homologous Recombination
HRAS: Harvey Rat Sarcoma Viral Oncogene Homolog
HSPGs: Heparan Sulfate Proteoglycans
IFN-α, IFN-β: Interferon Alpha, Interferon Beta
IL-1β: Interleukin-1 Beta
IRF: Interferon Regulatory Factor
ISGF3: Interferon-stimulated Gene Factor 3
JUN: Jun Proto-Oncogene, AP-1 Transcription Factor Subunit
KDM5C, 6A: Lysine Demethylase 5C, 6A
KLHL6: Kelch-like Family Member 6
KLF: Kruppel-Like Factor
KRAS: Kirsten Rat Sarcoma Viral Oncogene Homolog
Ku70-80: X-Ray Repair Cross-Complementing Protein 6–5
LCR: Long Control Region
LEPREL1: Leprecan-Like Protein 1
LPP: LIM Domain Containing Preferred Translocation Partner In Lipoma
LKB1: Liver Kinase B1 (Serine/threonine kinase 11—STK11)
LRP1B: LDL Receptor-related Protein 1B
MACROD2: MACRO Domain Containing 2
MAML: Mastermind Like Transcriptional Coactivator 1
MAPK (ERK): Mitogen-Activated Protein Kinase
MCM: Minichromosome Maintenance
MEK/ERK: Mitogen-activated Protein Kinase/Extracellular signal-Regulated Kinase
MHC: Major Histocompatibility Complex
miRNA: MicroRNA
MLL2, MLL3: Mixed Lineage Leukemia 2, Mixed Lineage Leukemia 3
MRE11: Meiotic Recombination 11 Homolog
MRN: MRE11-RAD50-NBS1 complex
mTOR: Mammalian Target of Rapamycin
MYBL2: MYB Proto-Oncogene Like 2
MYC: MYC Proto-Oncogene, BHLH Transcription Factor
NBS1: Nijmegen Breakage Syndrome 1
NFE2L2: Nuclear Factor Erythroid 2–related Factor 2
NF-κB: Nuclear Factor kappa-light-chain-enhancer of activated B cells
NFX1-91: Nuclear Transcription Factor, X-Box Binding 1
NGFR: Nerve Growth Factor Receptor
NHEJ: Non-Homologous End Joining
NOL7: Nucleolar Protein 7
NOTCH1: Neurogenic Locus Notch Homolog Protein 1
NR4A2: Nuclear Receptor Subfamily 4 Group A Member 2
NuMA: Nuclear Mitotic Apparatus Protein 1
OPSCC: Oropharyngeal Squamous Cell Carcinoma
ORF: Open Reading Frame
PALB2: Partner and Localizer of BRCA2
PAXX: PAXX Non-Homologous End Joining Factor
PBM: PDZ-Binding Motif
PD1, PDL1, PDL2: Programmed Death 1, Programmed Death Ligand 1, Programmed Death Ligand 2
PE: Early Promoter
PI3K/AKT: Phosphoinositide 3-kinase/Protein Kinase B
PLGRKT: Plasminogen Receptor with a C-terminal Lysine
PLK1: Polo Like Kinase 1
POLη: Polymerase Eta
POU5F1B: POU Class 5 Homeobox 1B
PRC: Polycomb Repressive Complex
PTEN: Phosphatase And Tensin Homolog
RAD9: RAD9 Checkpoint Clamp Component
RAD51: RAD51 Recombinase
RB (RB1): Retinoblastoma Protein (Retinoblastoma 1 gene)
RICTOR: Rapamycin-insensitive Companion of mTOR
RIF1: Replication Timing Regulatory Factor 1
RIPK4: Receptor Interacting Serine/Threonine Kinase 4
RNF168: Ring Finger Protein 168
RPA: Replication Protein A
RTK-JAK-STAT: Receptor Tyrosine Kinase-Janus Kinase-Signal Transducer and Activator of Transcription
SDSA: Synthesis-Dependent Strand Annealing
SEMA3D: Semaphorin 3D
SHKBP1: SH3KBP1 Binding Protein 1
SMC1: Structural Maintenance Of Chromosomes 1
SMAD2/3, SMAD4: Mothers Against Decapentaplegic Homolog 2/3, Mothers Against Decapentaplegic Homolog 4
SNX27: Sorting Nexin 27
SOX2: SRY-box Transcription Factor 2
SP1: Sp1 Transcription Factor
SSB: Single-Stranded DNA Break
STAT1: Signal Transducer And Activator Of Transcription 1
STING: Stimulator of Interferon Genes
STR: BLM-TOPOIIIα-RMI1-RMI2 complex
TBP: TATA-box Binding Protein
TERT: Telomerase Reverse Transcriptase
TGF-β: Transforming Growth Factor Beta
TGFBR2: Transforming Growth Factor Beta Receptor 2
TIP60: Tat Interacting Protein 60
TLS: Translesion Synthesis
TLR9: Toll-like Receptor 9
TNFα, TNFR1: Tumor Necrosis Factor Alpha, Tumor Necrosis Factor Receptor 1
TNM: Tumor, Node, Metastasis
TOPBP1: DNA Topoisomerase II Binding Protein 1
TP53, TP63: Tumor Protein p53, p63
TPRX1: Tetrapeptide Repeat Homeobox 1
TRAF3: TNF Receptor Associated Factor 3
TSC2: Tuberous Sclerosis Complex 2
Tyk2: Tyrosine Kinase 2
UBR5: Ubiquitin Protein Ligase E3 Component N-Recognin 5
UICC: Union for International Cancer Control
URR: Upstream Regulatory Region
WRN: WRN RecQ Like Helicase
XLF: XRCC4-like factor
XRCC1,: X-Ray Repair Cross Complementing 1,
XRCC4: X-Ray Repair Cross Complementing 4
ZO-1: Zona Occludens 1
ZMIZ1: Zinc Finger MIZ-Type Containing 1
